# A New Way of Sealing Wounds

**Published:** 1903-01-24

**Authors:** 


### A NEW WAY OF SEALING WOUNDS.

ALTHOUGH we feel so satisfied with the practical value of the cyanide gauze which is now so generally used as a dressing for wounds that we are not likely to be easily led to put our trust in the claims of any other material for ordinary use, we are free to admit that cases occur in which the absolute sealing up of a wound may be desirable, and that in such cases the means hitherto at our disposal for this purpose have been far from satisfactory. Mr. Peyton Beale[1] has, however, drawn attention to a new "film" dressing which promises from his account to be of considerable service in such cases. This material is known as "velvril." It is a cellulose derivative which can be produced cheaply in the form of films of any desired thickness. It is transparent, but slightly yellow in colour; is absolutely impervious to all ordinary gases and fluids; and is tough, elastic, and very durable. It can be readily sterilised by any of the ordinary methods and can be immersed in boiling water for a long time without undergoing any permanent change. It can also be easily dissolved in acetone, and its solution in this fluid is quite unirritating, even when in contact with a freshly made wound, while the solution from the method of its manufacture is, we are told, absolutely sterile. In the use of this material the following method is adopted:—When the operation has been completed the skin and everything else having, of course, been rendered thoroughly aseptic, a piece of sterilised velvril film is cut large enough to cover the wound and the skin round it for at least 1 inch in each direction, and if a drain is to be used a sufficient opening is cut in the film to receive the tube or plug. The wound must be rendered as dry as possible, and if ordinary methods do not suffice to check capillary oozing washing the wound with 50 per cent. alcohol and water will be found very efficacious. When the wound has been sutured, it and the skin all round are washed freely with acetone, using dry wool for the purpose. The wound and skin are then painted freely with velvril solution, and the film is placed upon it, the exposed, that is the dry, surface being placed next the solution. Upon this is placed a pad of sterile wool and the whole is bandaged firmly, it being desirable to apply as much pressure as possible for the first 12 hours or so. The preliminary washing with acetone is necessary in order to remove all trace of water. The film thus applied sticks so firmly that it is absolutely impossible for any gas or fluid to gain access to the wound. If left to itself it will peel off in from 10 to 12 days, bringing the superficial layers of dead epithelial cells with it, but if the film has to be removed at any time it is only necessary to place a wet cloth or swab upon it for a few minutes, when, if one corner of the film be raised the whole can be peeled off with ease. If a drain is inserted it is as well that it should be placed at the lower end of the incision and that an absorbent sterile dressing should be placed over it, but a much better plan is to make a separate skin incision for the drainage tube or plug as far removed from the wound as possible. Judging from Mr. Peyton Beale's description, this material should offer to the surgeon many facilities in the closing of aseptic wounds. Obviously, however, we must have great confidence in their sterility before treating them in this manner.

[1] Lancet, Jan. 17.

